# Predictive modeling for mortality risk in elderly patients with pulmonary embolism using machine learning algorithms

**DOI:** 10.3389/fmed.2026.1879237

**Published:** 2026-08-05

**Authors:** Tao Chu, Ling Ji, Dingyu Tan, Huihui Wang, Qingcheng Zhu, Dan Dai

**Affiliations:** Department of Emergency Medicine, Northern Jiangsu People’s Hospital Affiliated to Yangzhou University, Yangzhou, China

**Keywords:** deep learning model, elderly patients, mortality, pulmonary embolism, risk stratification

## Abstract

**Background:**

Pulmonary Embolism (PE) is a condition that results in significant mortality and morbidity, particularly among the elderly. The aim of our study is to utilize machine learning (ML) algorithms specifically tailored for this population, thereby enhancing the accuracy of risk assessment.

**Methods:**

Elderly patients with PE who were included between January 1, 2013, and December 31, 2023, were divided into two groups: training and validation sets. A total of five ML models, including decision tree, random forest (RF), extreme gradient boosting, support vector machine, and k-nearest neighbors, were developed to predict in-hospital mortality in these patients. The model demonstrating the best diagnostic performance was selected. Ultimately, the ML models were internally validated to assess their diagnostic performance using receiver operating characteristic analysis.

**Results:**

The analysis included 250 patients, with 174 assigned to the training set and 76 to the validation set. The ML models were developed using nine clinical features: age, lactate levels, blood urea nitrogen, arterial oxygen partial pressure, red blood cell count, diastolic blood pressure, vasopressor use, acute kidney injury, and the requirement for continuous renal replacement therapy. Of the five evaluated ML models, the RF model demonstrated the best performance, attaining the highest area under the curve values of 0.950 for the training set and 0.835 for the validation set.

**Conclusion:**

ML-based models exhibit strong predictive capabilities for identifying elderly patients with PE who are at risk of hospital mortality. These algorithms can aid physicians in the early detection of high-risk patients, facilitating timely and appropriate preventive interventions. Future prospective studies comparing ML models with established PE-specific risk stratification tools are warranted.

## 1 Introduction

Pulmonary embolism (PE) is a potentially fatal condition characterized by nonspecific symptoms and signs (1). The annual incidence of PE is approximately one case per 1,000 individuals, resulting in an estimated 100,000–300,000 deaths each year (2). PE ranks as the third most common cause of cardiovascular disease events, following myocardial infarction and stroke (3). Early anticoagulation therapy has been shown to reduce the mortality rate from 30 to 8% (4). Consequently, identifying patients with acute PE who are at higher risk of mortality remains a significant challenge.

Numerous risk factors predispose patients to the development of PE, with age identified as an independent risk factor for both mortality and delayed diagnosis (5). According to a study by Pauley, all-cause inpatient mortality was highest among the oldest patients, with a rate of 2% among those aged 20–54 years and 11% among patients aged 85 years and older (6). Therefore, accurate risk stratification is particularly important in elderly patients with PE.

Various scoring systems, including the Pulmonary Embolism Severity Index (PESI), simplified PESI (sPESI), and the Sequential Organ Failure Assessment (SOFA) score, have been used for risk stratification in patients with PE (7). Previous studies have reported the area under the receiver operating characteristic curve (AUC) values of approximately 0.76–0.77 for PESI and sPESI in predicting short-term mortality among elderly patients with PE (8). Although these tools are clinically valuable and widely implemented, their predictive performance remains imperfect, indicating the potential need for approaches that can further improve individualized risk stratification in elderly patients with PE.

Machine learning (ML) is an emerging field that has significantly influenced clinical medical research. ML analysis employs advanced iterative algorithms to integrate candidate variables, thereby enabling accurate predictions (9). This advancement has spurred growing interest in applying ML techniques to medical diagnosis, treatment decisions, and genetic analysis (10). Notably, ML-based prediction models have shown promising performance in critical care medicine. Ong et al. demonstrated that ML models outperformed conventional statistical models in predicting clinical outcomes among patients receiving mechanical ventilation in the intensive care unit (ICU) (11). Likewise, Zhou et al. developed an interpretable ML model for predicting 28-day mortality in patients with sepsis-induced coagulopathy, which achieved superior discrimination compared with traditional severity scores and maintained robust performance across multiple external validation cohorts (12).

More recently, ML techniques have also been applied to PE. Jenab et al. developed ML models for predicting adverse clinical outcomes in hospitalized patients with PE, while Wang et al. constructed an extreme gradient boosting (XGBoost)-based model for mortality prediction in critically ill patients with PE (2, 13). However, these studies mainly included general or mixed PE populations and did not specifically focus on elderly patients admitted to the ICU. Nonetheless, there exists a deficiency in comprehensive models explicitly tailored for older patients suffering from PE to forecast in-hospital mortality.

Thus, our objective is to utilize ML techniques to identify the risk factors associated with hospital mortality in elderly patients suffering from PE and to develop a predictive model based on patient status. The proposed model aims to assist clinicians in tailoring personalized intervention strategies, ultimately enhancing patient outcomes.

## 2 Patients and methods

### 2.1 Study design and patients

This retrospective study was carried out in a 72-bed ICU at a tertiary teaching hospital in Jiangsu Province, China. The study adhered to the ethical guidelines set forth by the revised Declaration of Helsinki. Approval was obtained from the Institutional Ethics Committee of Northern Jiangsu People’s Hospital (No. 20241251). Due to the retrospective nature of the study, the requirement for written informed consent was waived.

In this study, we retrospectively collected clinical data from all hospitalized patients diagnosed with PE between January 1, 2013, and December 31, 2023. The diagnostic criteria for PE were based on the most recent guidelines published by the European Society of Cardiology (ESC) regarding the diagnosis and management of PE (14), primarily relying on imaging confirmation through computed tomography pulmonary angiography. Participants in this study had to be at least 65 years old and have complete laboratory and clinical data available within 24 h of admission. The exclusion criteria consisted of end-stage chronic diseases with a life expectancy of less than 3 months, a history of malignant tumors, and chronic PE.

### 2.2 Variable extraction

Information was gathered from electronic health records, including demographic information such as gender, age, height, weight, and medical history. Vital signs upon admission were documented, particularly the respiratory rate, heart rate, systolic blood pressure, and diastolic blood pressure (DBP). Additionally, the SOFA scores were assessed upon admission. Laboratory test indicators of patients’ blood were also collected at this stage. Table 1 shows detailed data. Furthermore, it was noted whether patients required invasive ventilatory support, the administration of vasopressors upon hospital admission, the occurrence of acute kidney injury (AKI) during treatment, and whether continuous renal replacement therapy (CRRT) was necessary.

**TABLE 1:** Baseline characteristics in training and validation sets.

Variables	Training set			Validation set			
	Survived (*n* = 117)	Died (*n* = 57)	*P* (Intra)	Survived (*n* = 51)	Died (*n* = 25)	*P* (Intra)	*P* (Inter)
Male, n (%)	49 (41.9)	29 (50.9)	0.338	27 (52.9)	15 (60.0)	0.737	0.167
Age, years	68 (65–75)	74 (69–80)	0.001	68 (64–73)	77 (75–81)	< 0.001	0.469
Height (cm)	172 (165–178)	168 (160–173)	0.031	173 (157–178)	168 (165–175)	0.965	0.855
Weight (kg)	76.7 (67.8–95.6)	85.5 (67.5–100.0)	0.275	80.0 (66.2–96.7)	77.5 (67.0–91.0)	0.558	0.464
BMI (kg/m^2^)	27.1 (24.0–31.7)	29.7 (26.1–32.9)	0.135	28.0 (23.6–32.9)	26.3 (23.5–31.8)	0.651	0.375
Comorbidities, n (%)							
COPD	25 (21.4)	14 (24.6)	0.779	14 (27.5)	2 (8.0)	0.098	0.942
Coronary artery disease	32 (27.4)	11 (19.3)	0.333	16 (31.4)	4 (16.0)	0.249	0.912
Hypertension	43 (36.8)	21 (36.8)	1.000	19 (37.3)	12 (48.0)	0.518	0.646
Diabetes mellitus	32 (27.4)	19 (33.3)	0.525	9 (17.6)	8 (32.0)	0.264	0.327
Sepsis	90 (76.9)	48 (84.2)	0.361	37 (72.5)	22 (88.0)	0.220	0.896
Vital signs							
Respiratory rate (/min)	20 (16–26)	23 (17–27)	0.183	19 (16–24)	26 (22–28)	0.003	0.670
Heart rate (/min)	91 (79–107)	98 (87–114)	0.108	88 (78–114)	100 (77–125)	0.416	0.664
Systolic blood pressure (mmHg)	118 (100–131)	109 (91–125)	0.082	119 (100–136)	118 (109–137)	0.603	0.132
Diastolic blood pressure (mmHg)	61 (52–71)	61 (49–69)	0.291	62 (55–73)	62 (51–68)	0.514	0.760
SOFA	6 (4–9)	10 (7–13)	< 0.001	6 (4–9)	8(5–12)	0.05	0.415
Laboratory data							
Red blood cell (*10^12^)	3.87 (3.44–4.28)	3.56 (2.89–4.10)	0.006	3.76 (3.02–4.33)	3.69 (3.33–4.16)	0.978	0.570
White blood cell (*10^9^)	10.9(8.4–15.1)	13.9 (8.0–19.8)	0.101	12.1 (8.9–16.7)	10.6 (9.2–14.1)	0.476	0.737
Platelet (*10^9^)	197 (144–279)	185 (111–284)	0.311	213 (151–289)	170 (135–232)	0.167	0.854
Hemoglobin (g/L)	114 (99–130)	108 (89–122)	0.044	112 (91–130)	113 (99–119)	0.703	0.349
Hematocrit (%)	35.2 (31.8–39.7)	33.7 (27.6–39.4)	0.155	34.3(27.9–39.9)	35.5(32.0–37.1)	0.791	0.401
Total bilirubin (mg/dL)	0.6 (0.5–1.0)	0.7 (0.5–1.4)	0.089	0.7 (0.4–0.9)	0.7(0.4–1.2)	0.598	0.263
Alanine aminotransferase (U/L)	23 (16–50)	27 (19–74)	0.116	23 (13–42)	27(20–124)	0.060	0.661
Aspartate aminotransferase (U/L)	35 (21–66)	59 (28–122)	0.005	32 (22–43)	46 (28148)	0.028	0.477
Lactate dehydrogenase (U/L)	282 (197–404)	370 (262–643)	< 0.001	289 (229–446)	406 (314–598)	0.017	0.356
Sodium (mmol/L)	138 (136–141)	139 (135–142)	0.241	139 (136–142)	140 (137–142)	0.587	0.179
Potassium (mmol/L)	4.2 (3.8–4.8)	4.40 (3.7–5.1)	0.464	4.2 (3.7–4.8)	4.1(3.7–4.4)	0.288	0.320
Calcium (mmol/L)	2.1(2.0–2.3)	2.1(2.0–2.2)	0.581	2.0 (1.8–2.2)	2.2 (1.90–2.3)	0.070	0.008
Glucose (mmol/L)	7.0(5.8–10.3)	7.9(6.5–13)	0.026	9.4(6.6–11.7)	12.3(7.7–14.3)	0.088	0.446
Creatinine (mg/dL)	1.0(0.8–1.5)	1.3(0.9–1.9)	0.020	0.9 (0.8–1.2)	1.3 (0.9–1.6)	0.046	0.299
Blood urea nitrogen (mmol/L)	22(16–36)	29 (21–45)	0.014	20 (15–27)	28 (21–44)	0.005	0.213
Albumin (g/dL)	2.9 (2.6–3.3)	2.9 (2.4–3.4)	0.631	2.7 (2.5–3.2)	2.9 (2.6–3.4)	0.409	0.430
Prothrombin time (seconds)	14.2 (12.6–18.0)	15.6 (13.9–20.8)	0.026	14.2(12.5–17.0)	18.0(13.9–24.9)	0.023	0.676
APTT (seconds)	32.3 (27.5–44.3)	35.2(29.6–48.4)	0.211	35.8 (28.4–61.1)	35.4(28.6–45.4)	0.514	0.298
INR	1.3(1.1–1.7)	1.4(1.3–1.9)	0.011	1.3 (1.1–1.6)	1.4 (1.3–2.0)	0.036	0.860
Arterial pH	7.37 (7.30–7.43)	7.35 (7.21–7.40)	0.091	7.38 (7.29–7.43)	7.37 (7.27–7.41)	0.615	0.579
PaO_2_ (mmHg)	108 (69–230)	85(70–162)	0.168	122 (76–191)	98(84–123)	0.325	0.795
PaCO_2_ (mmHg)	40(35–47)	39 (31–47)	0.290	39 (36–44)	37(34–43)	0.298	0.580
Lactic acid (mmol/L)	1.9(1.3–3.4)	2.9 (2.0–7.5)	< 0.001	1.8(1.3–3.3)	2.6(1.7–5.9)	0.056	0.208
Invasive ventilator, n (%)	88 (75.2)	48 (84.2)	0.249	37 (72.5)	23 (92.0)	0.098	1.000
Vasopressor, n (%)	72 (61.5)	52 (91.2)	< 0.001	27(52.9)	22 (88.0)	0.006	0.357
Acute kidney injury, n (%)	41 (35.0)	33 (57.9)	0.007	21 (41.2)	12 (48.0)	0.751	1.000
CRRT, n (%)	10 (8.5)	13 (22.8)	0.018	3 (5.9)	4 (16.0)	0.312	0.493

COPD, Chronic obstructive pulmonary disease; SOFA, Sequential Organ Failure Assessment; APTT, Activated partial thromboplastin time; INR, International normalized ratio; PaO_2_, arterial oxygen partial pressure; PaCO_2_, arterial carbon dioxide partial pressure; CRRT, Continuous renal replacement therapy.

### 2.3 Data pre-processing

Initially, candidate variables with missing rates greater than 10% were planned for exclusion. However, no variable exceeded this threshold in the present study. Missing values among retained predictors were handled using Multiple Imputation by Chained Equations (MICE) prior to model development. Variables without missing values were analyzed directly. Detailed missing-data proportions and handling procedures for all candidate predictors are presented in Supplementary Table 1. Additionally, categorical data were converted into dummy variables for subsequent model construction. The final set of input features comprised 41 clinical characteristics of the patients.

In order to identify a subset of features that maximizes information content, the least absolute shrinkage and selection operator (LASSO) regression technique was applied to determine the most important predictors of mortality in hospitals. To reduce bias during subsequent model training and to improve the interpretability of the developed machine learning model, the variance inflation factor (VIF) was used to evaluate multicollinearity among the chosen features, ensuring that the VIF values for the features employed in model training stayed below 5.

### 2.4 Model development and assessment

The dataset was randomly divided into a training group comprising 70% of the data and an independent validation group constituting 30%. Model development involved trials with five machine learning algorithms: decision tree (DT), random forest (RF), XGBoost, support vector machine (SVM), and k-nearest neighbor (KNN). During model development, 10-fold cross-validation was employed for model training and hyperparameter tuning. Grid-search optimization was used to identify the optimal parameter combinations for each ML algorithm. After identifying the best-performing model, additional internal validation procedures were conducted. Calibration curves were generated to assess the agreement between predicted and observed mortality probabilities, while bootstrap resampling (1,000 repetitions) was performed to evaluate model stability and estimate optimism-corrected predictive performance.

To assess the predictive capabilities of each model, we generated receiver operating characteristic (ROC) curves, which acted as our main evaluation metric. Furthermore, we calculated decision curve analysis (DCA) curves, as well as metrics including precision, recall, accuracy, F1 score, negative predictive value (NPV), and positive predictive value (PPV). The best-performing ML model was further compared with the SOFA score. We selected SOFA as a comparator because it is a widely used ICU severity assessment tool and was consistently available for all included patients. PE-specific risk scores such as PESI and sPESI could not be reliably calculated because peripheral oxygen saturation (SpO_2_) and complete information on a history of chronic cardiopulmonary disease, both required for sPESI calculation, were not consistently available in the retrospective dataset. Similarly, ESC risk classification could not be fully applied because right ventricular assessment and cardiac biomarker data were incompletely documented.

Differences between the AUCs were compared using the DeLong test. To account for multiple comparisons between ML models and the SOFA score, Bonferroni correction was applied where appropriate.

### 2.5 Feature interpretation

The shapley additive explanation (SHAP) method was employed to elucidate the key features that significantly contributed to the predictions made by the trained ML model. Specifically, the SHAP algorithm computes the shapley value for each feature, representing the average contribution of that feature to the prediction across all possible combinations of features. In addition, SHAP dependence plots were constructed for the most important features to further explore the relationships between feature values and their corresponding contributions to the predicted risk.

### 2.6 Statistical analysis

The Kolmogorov-Smirnov test was employed to evaluate the normality of measurement data. Data that followed a normal distribution were presented as means ± standard deviations, while skewed data were reported using medians and quartiles. For group comparisons, *t*-tests or Mann-Whitney U tests were utilized, and categorical variables were analyzed using chi-square tests or Fisher’s exact tests. To account for multiple comparisons in model performance evaluation, *P*-values were adjusted using the Bonferroni correction method where appropriate. Statistical analyses were carried out with R (version 3.6.1) and SPSS (version 24.0), setting the significance level at *P* < 0.05.

## 3 Results

### 3.1 Baseline characteristics and outcomes

**FIGURE 1:**
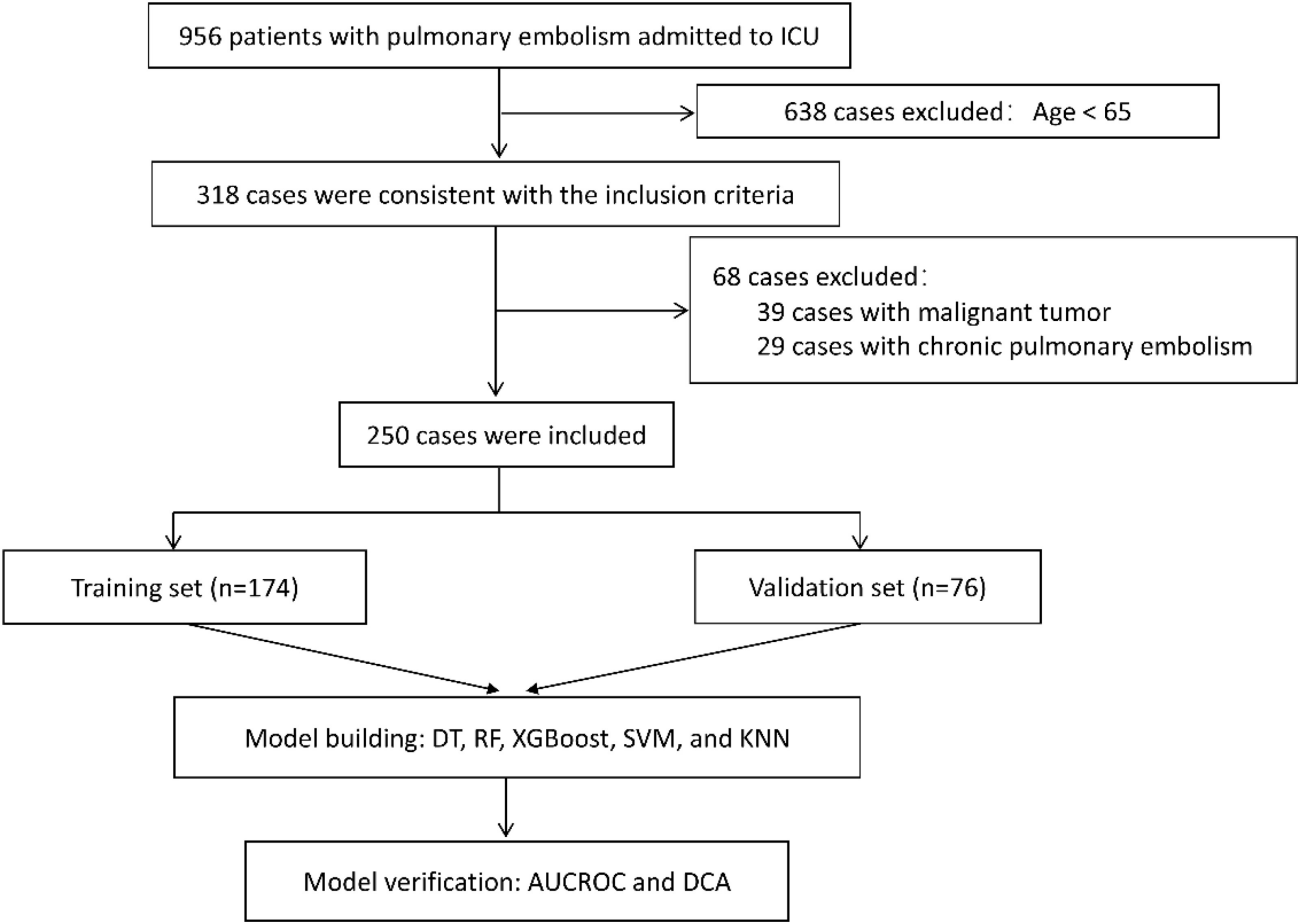
Flowchart of the research process. ICU, intensive care unit. DT, decision tree. RF, random forest. XGBoost, extreme gradient boosting. SVM, support vector machines. KNN, k-nearest neighbor. AUROC, area under the receiver operating characteristic curve. DCA, decision curve analysis.

During the study period, of the 318 elderly patients with PE who met the inclusion criteria, 68 were excluded for the following reasons: 39 with a history of malignant tumors, and 29 due to chronic PE. Consequently, 250 patients were included in the analysis, comprising 174 in the training set and 76 in the validation set (Figure 1). The in-hospital mortality rate in our study was 32.80%, with the training and validation sets exhibiting mortality rates of 32.76 and 32.89%, respectively.

Between the training and validation sets, there were no significant differences in basic characteristics, except for blood calcium concentrations (Table 1). In the training cohort, statistically significant differences were observed between the survival group and the deceased group regarding age, height, SOFA score, red blood cell (RBC), hemoglobin levels, aspartate aminotransferase, lactate dehydrogenase, glucose, creatinine, blood urea nitrogen (BUN), prothrombin time, international normalized ratio, arterial oxygen partial pressure (PaO_2_), lactate, the necessity for vasopressor administration, the presence of AKI, and the requirement for CRRT (Table 1).

P (Intra) represents the comparison between survivors and non-survivors within each cohort. P (Inter) represents the comparison of baseline characteristics between the training and validation cohorts.

### 3.2 Predictive feature selection

**FIGURE 2:**
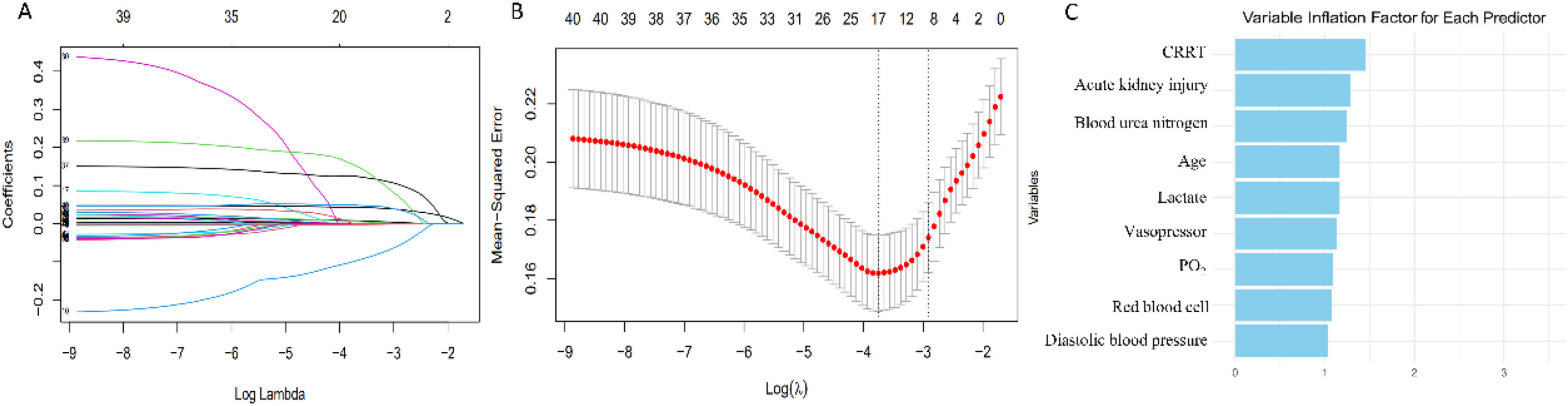
Identification of the risk factors of hospital mortality by least absolute shrinkage and selection operator (LASSO) regression. **(A)** LASSO coefficient profiles of the 41 variables. **(B)** The cross-validation curves for LASSO regression are presented, with the y-axis representing binomial deviation. The dashed line on the left indicates the log(λ) at which the model deviation is minimized, while the dashed line on the right represents the log(λ) at the minimum model deviation plus one standard error. We selected the log(λ) and the corresponding variables associated with the dashed line on the right. **(C)** The variance inflation factor results of the selected features.

In the training set, the variables identified were further analyzed using LASSO binary logistic regression, with the lambda value selected according to the 1-standard-error criterion (Figures 2A,B). Nine independent risk factors for mortality were identified: CRRT, AKI, BUN, age, lactate levels, vasopressor use, PaO_2_, RBC, and DBP. After assessing for multicollinearity, it was determined that all included indicators had VIF values below 5, indicating the absence of collinearity issues, as illustrated in Figure 2C.

### 3.3 Predictive models

Following the predictive feature selection, we evaluated the performance of the models by integrating the variables into five ML models and visualizing their features using AUC. Table 2 presents seven metrics for assessing model performance, which include AUC, precision, recall, accuracy, F1 score, NPV, and PPV.

**TABLE 2:** Discrimination indicators of the predictive models in the training and validation sets.

Variables	AUC	Precision	Recall	Accuracy	F1 Score	NPV	PPV
Training set							
Decision tree	0.761	0.935	0.509	0.828	0.659	0.804	0.935
Random Forest	0.950	0.831	0.860	0.897	0.845	0.930	0.831
XGBoost	0.863	0.677	0.772	0.805	0.721	0.881	0.677
SVM	0.875	0.800	0.702	0.845	0.748	0.863	0.800
KNN	0.861	0.662	0.825	0.805	0.734	0.903	0.662
SOFA	0.723	0.477	0.719	0.649	0.573	0.818	0.477
Validation set							
Decision Tree	0.665	0.667	0.320	0.724	0.432	0.734	0.667
Random forest	0.835	0.625	0.600	0.750	0.612	0.808	0.625
XGBoost	0.813	0.654	0.680	0.776	0.667	0.840	0.654
SVM	0.833	0.789	0.600	0.816	0.682	0.825	0.789
KNN	0.738	0.567	0.680	0.724	0.618	0.826	0.567
SOFA	0.639	0.417	0.600	0.592	0.492	0.750	0.417

AUC, Area under the receiver operating characteristic curve; NPV, Negative predictive value; PPV, Positive predictive value; XGBoost, Extreme gradient boosting; SVM, Support vector machine; KNN, K-nearest neighbor; SOFA, Sequential Organ Failure Assessment.

**FIGURE 3:**
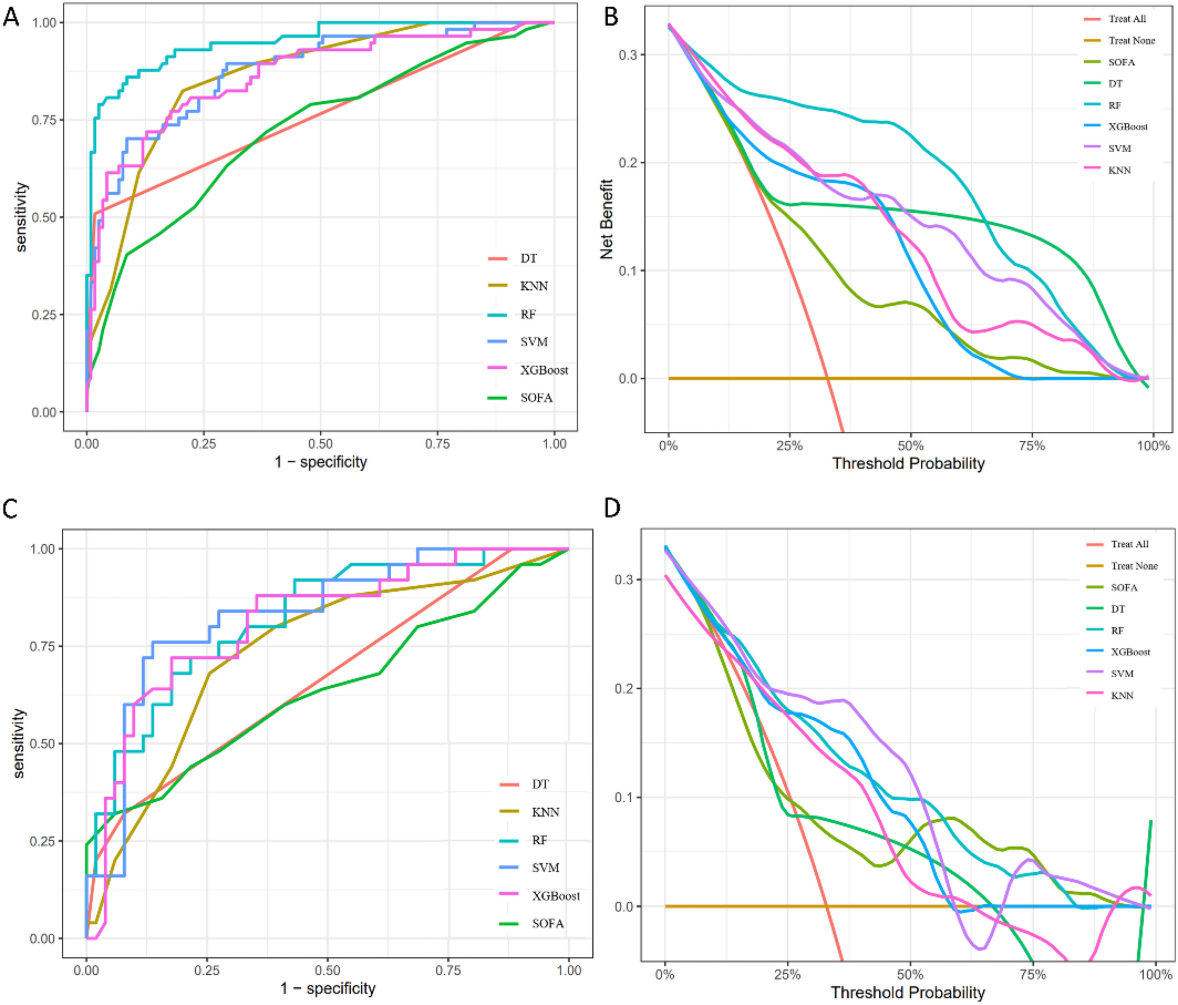
**(A)** Receiver operating characteristic (ROC) curves for different models and the Sequential Organ Failure Assessment (SOFA) score in predicting hospital mortality in the training set. **(B)** Decision curves (DCA) for different models and SOFA score in the training set. **(C)** ROC curves for different models and SOFA score in predicting hospital mortality in the validation set. **(D)** DCA curves for different models and SOFA score in the validation set.

In the training set, the RF model achieved the highest predictive performance, with an AUC of 0.950, recall of 0.860, accuracy of 0.897, F1 score of 0.845, and NPV of 0.930. Other models demonstrated moderate predictive ability, while the DT model performed less optimally. All ML models demonstrated better discrimination than the SOFA score in this cohort (Figure 3A). The RF model demonstrated significantly better discrimination than the SOFA score (unadjusted *P* = 0.001). After Bonferroni correction for multiple comparisons, the difference remained statistically significant (adjusted *P* = 0.005). Decision curve analysis (DCA) further confirmed that the RF model provided the greatest net benefit across a wide range of risk thresholds (Figure 3B).

In the validation set, the RF model maintained robust discrimination (AUC = 0.835), confirming its generalizability. The performance of other models was consistent with the training set, and all ML models still outperformed the SOFA score (Figure 3C). DCA curves in the validation set similarly indicated that the RF model offered the highest net clinical benefit (Figure 3D).

These results demonstrate that the RF model is the most reliable among the evaluated algorithms for predicting hospital mortality in elderly patients with PE, providing both superior discrimination and potential clinical utility.

### 3.4 Calibration and internal validation of the optimal model

**FIGURE 4:**
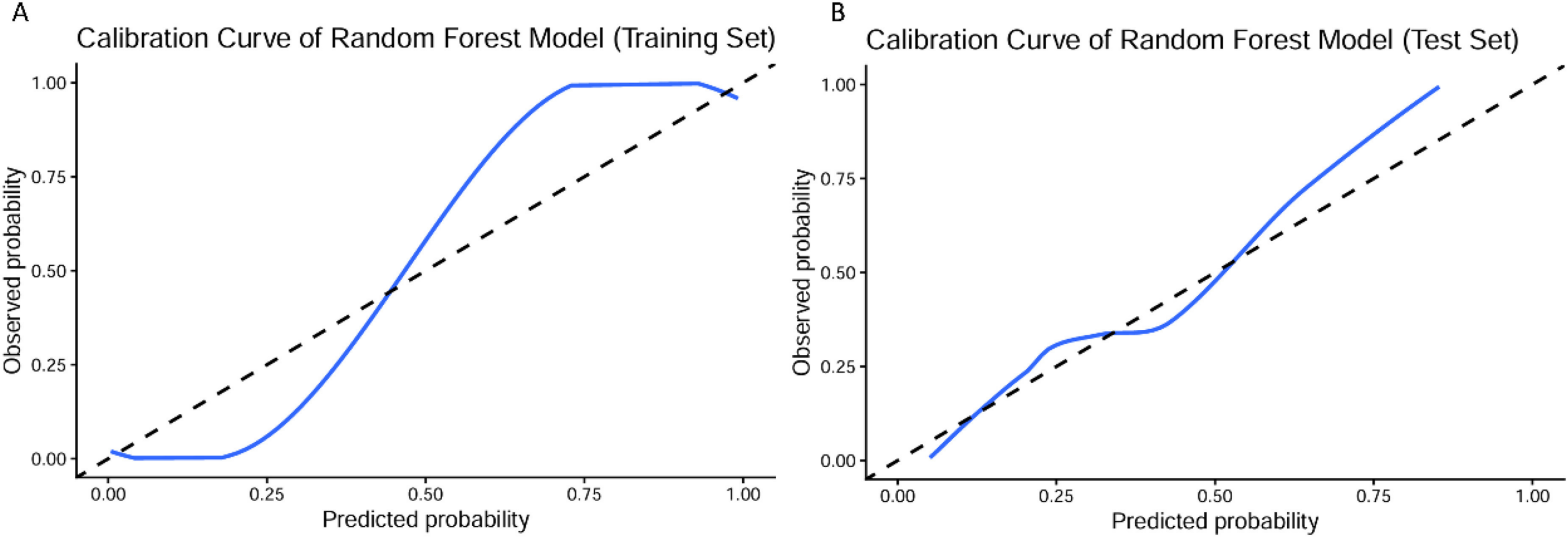
Calibration curves of the random forest model in the training **(A)** and validation **(B)** cohorts.

To further evaluate the reliability of the optimal RF model, calibration analysis and bootstrap validation were performed. The calibration curve demonstrated good agreement between predicted and observed mortality probabilities, indicating satisfactory calibration performance (Figure 4).

Bootstrap resampling with 1,000 repetitions was conducted to assess model stability and quantify optimism. The apparent AUC of the RF model was 0.950, whereas the optimism-corrected AUC was 0.923, corresponding to an optimism estimate of 0.027 (Supplementary Table 2). These findings suggest limited overfitting and support the robustness of the model.

### 3.5 Model interpretation and optimization

**FIGURE 5:**
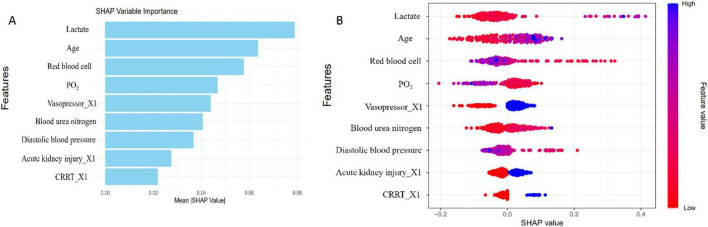
The shapley additive explanation (SHAP) analysis results for the optimal hospital mortality prediction model utilizing random forest. **(A)** Average impact of each clinical feature on model output magnitude. **(B)** SHAP summary plot of selected variables. Each variable of each patient was color-coded by a point according to an attribute value.

Presented in Figure 5 is the ranking of SHAP values corresponding to each feature within the training dataset, derived from the optimal predictive model of RF. The findings demonstrate that lactate levels emerged as the most influential factor affecting the prediction of hospital mortality among elderly patients suffering from PE, succeeded by age, RBC, PaO_2_, vasopressor usage, and several other factors. Additionally, it was found that lower lactate levels, diminished BUN levels, a younger age, absence of vasopressor administration, lack of AKI, as well as a non-requirement for CRRT correlate with reduced mortality. On the other hand, decreased levels of RBC, PaO_2_, and DBP were linked to a heightened risk of mortality.

To further explore the relationships between key predictors and model output, SHAP dependence plots for the four most influential variables (Lactate, age, RBC, and PaO_2_) were generated and are presented in Supplementary Figure 1. The plots demonstrated nonlinear associations between these variables and mortality risk. Higher age and lactate levels were associated with progressively increased SHAP values, indicating a greater contribution to predicted mortality, whereas higher RBC and PaO_2_ levels were generally associated with lower SHAP values, suggesting a protective effect.

## 4 Discussion

PE is a prevalent and life-threatening condition among emergency patients, particularly affecting the elderly (15). To our knowledge, studies specifically evaluating and comparing multiple ML algorithms for in-hospital mortality prediction in elderly ICU patients with PE remain limited. In this study, we identified nine independent prognostic factors for in-hospital mortality in this population: CRRT, AKI, BUN, age, lactate levels, vasopressor use, PaO_2_, RBC, and DBP. Different ML models exhibited favorable predictive performance for in-hospital mortality and demonstrated better discrimination than the SOFA score in this cohort. Among the five ML models evaluated, the RF model emerged as the optimal choice, achieving the highest AUC of 0.950 in the training set and an AUC of 0.835 in the validation set. Although ML models are more complex than conventional scoring systems, the RF model demonstrated superior discrimination and good calibration, supporting its potential value for risk assessment in elderly ICU patients with PE.

Currently, several PE-specific risk stratification tools have been developed and validated. Among them, the PESI and sPESI are the most widely used clinical prediction models (16, 17). Zwierzina et al. demonstrated that both PESI and sPESI provided good prognostic performance in elderly patients with PE, with AUC values of approximately 0.76–0.77 for short-term mortality prediction (8). In addition, the current ESC guidelines recommend risk stratification based on hemodynamic status, right ventricular dysfunction, and cardiac biomarkers (14). More recently, novel tools such as PE-SCORE have been proposed to improve prediction of short-term clinical deterioration in patients with acute PE (18). These established risk assessment strategies provide important benchmarks for evaluating emerging ML approaches.

ML has emerged as a promising tool for critical care risk assessment because it can capture complex nonlinear relationships among clinical variables (19). Previous studies have shown that ML-based models may improve outcome prediction compared with conventional severity scores. For example, Deng et al. demonstrated improved ICU mortality prediction using a long short-term memory model (20), and Yuan et al. reported robust performance of a LightGBM model in elderly neurocritical patients (21). Building upon this evidence, we developed and validated ML models specifically for elderly patients with PE. Among the evaluated algorithms, the RF model achieved the best discrimination. Compared with the SOFA score, which served as an ICU severity assessment tool rather than a PE-specific prognostic model, the RF model demonstrated improved predictive performance in our cohort. These findings suggest that ML-based approaches may provide incremental prognostic value for individualized risk stratification in elderly patients with PE. However, because ML models generally require electronic health record integration and computational support, their principal advantage lies in improving predictive accuracy rather than simplifying clinical workflows.

Several ML-based prediction models for patients with PE have been reported in recent years. For example, Jenab et al. developed ML models to predict adverse clinical outcomes in hospitalized patients with PE, with the Gradient Boosting model achieving an AUC of 0.94, outperforming both deep learning and logistic regression models (2). Similarly, Wang et al. constructed an XGBoost-based model for predicting 30-day mortality in critically ill patients with PE, reporting a validation AUC of 0.82, which also outperformed the sPESI score (13). However, these studies primarily included general or mixed PE populations and were not specifically designed for elderly ICU patients. Given the unique clinical characteristics, higher comorbidity burden, and increased mortality risk in elderly patients with PE, a dedicated prediction model for this population may be particularly valuable. Our study extends the existing literature by focusing specifically on elderly patients with PE admitted to the ICU and by incorporating SHAP-based interpretation to improve model transparency and clinical applicability.

The predictive capabilities of various ML models remain a topic of debate. Notably, the RF model has been employed in the diagnosis of multiple diseases. It integrates numerous regression trees utilizing bootstrap aggregation and randomization of predictors to attain high predictive accuracy (22). In a study conducted by Yang et al., the RF model demonstrated superiority over other ML models, achieving an AUC of 0.787 for predicting cardiovascular disease (23). Additionally, Emir found that the RF model effectively screens patients who may require more comprehensive examinations for fibromyalgia (24). Similarly, our findings indicate that the RF model outperforms other ML models, providing an accurate and comprehensive tool for predicting hospital mortality in elderly patients with PE.

Elderly patients not only face an increased risk of PE incidence, but also experience a heightened risk of adverse clinical outcomes, including PE recurrence, anticoagulation-related bleeding, and mortality (25). The typical symptoms and signs of PE are more prevalent in patients without underlying cardiopulmonary diseases; however, the clinical manifestations can differ in elderly patients (26). Retrospective studies indicate that older patients with PE are more likely to experience syncope and report lower rates of chest pain compared to their younger counterparts (27). In older adults who possess a reduced organ reserve, a persistent inflammatory condition frequently occurs, leading to an increased rate of negative outcomes (28). Age was among the most influential variables identified by the RF model, consistent with previous studies demonstrating an association between advanced age and adverse outcomes in PE.

Elevated lactate has been identified as a key predictor of mortality in PE, a finding corroborated by numerous prior studies. In a prospective study involving 419 critically ill patients, lactate levels exceeding the upper limit of normal were associated with an increased risk of mortality (29). Lactate serves as a marker for tissue perfusion adequacy and has been shown to correlate with disease severity across various shock states, including sepsis, trauma, and PE (30). Additionally, recent studies have demonstrated that RBC is associated with the prediction, severity, and prognosis of PE. The underlying biomolecular mechanisms linking RBC to PE remain largely unexplored; however, it is hypothesized that anisocytosis may increase blood viscosity, leading to more stagnant blood flow (31). This stagnation could, in turn, enhance blood cell contact with endothelial walls, potentially triggering thrombosis through increased platelet and fibrin activation (32). In our research, lactate, age, and RBC were ranked among the top three factors significantly influencing mortality in elderly patients with PE. Therefore, regular monitoring of lactate levels and RBC in elderly PE patients is essential for effective management of their condition.

However, our study has several limitations. Firstly, it is retrospective in nature, which raises the possibility of selection and detection bias; thus, prospective studies are warranted to enhance the level of evidence. In addition, information regarding Do-Not-Intubate or Do-Not-Resuscitate orders was not available in our retrospective dataset, and these patients could not be identified separately. However, all included patients received active ICU management, and those with anticipated death within 24 h of ICU admission were excluded. Secondly, although the RF model demonstrated strong discrimination and calibration in both the training and validation cohorts, external validation using independent multicenter datasets is still required to confirm its generalizability. Thirdly, although our predictive model demonstrated superior performance compared with the SOFA score, direct comparison with established PE-specific risk stratification tools (e.g., PESI and sPESI) was not feasible because SpO_2_ and complete documentation of chronic cardiopulmonary disease history were not consistently available for reliable retrospective sPESI calculation. In addition, ESC risk classification could not be fully performed because right ventricular assessment and cardiac biomarker data were unavailable in a substantial proportion of patients. Although multiple strategies were applied to reduce overfitting and optimism bias, including feature selection, cross-validation, calibration analysis, and bootstrap validation, the relatively limited sample size and number of outcome events may still affect model stability and generalizability. This limitation should be addressed in future prospective studies. Finally, given the “black-box” characteristic of machine learning, future efforts should focus on enhancing model visualization, thereby facilitating its clinical utility through web-based platforms.

## 5 Conclusion

In this study, we employed ML algorithms to develop and validate models for predicting hospital mortality among elderly patients with PE. These algorithms have the potential to assist physicians in identifying high-risk patients earlier, thereby enabling timely and appropriate preventive measures. Future prospective multicenter studies comparing ML models with established PE-specific risk stratification tools, including PESI, sPESI, and ESC risk classification, are warranted to further evaluate their clinical utility.

## Data Availability

The raw data supporting the conclusions of this article will be made available by the authors, without undue reservation.
