# Supplementary material for: Predictive modeling for mortality risk in elderly patients with pulmonary embolism using machine learning algorithms
Source: Front Med (Lausanne). 2026 Aug 5;13:1879237. doi: 10.3389/fmed.2026.1879237 (PMC13485903; doi:10.3389/fmed.2026.1879237)
Supplement: Supplementary file 1 [file Table_1.docx]

**Supplementary Table S1.** Missing-data proportions and imputation strategy for candidate predictors.

| Variable | Missing n (%) | Handling method |
| --- | --- | --- |
| Male, n (%) | 0 | Complete |
| Age, years | 0 | Complete |
| Height (cm) | 24(9.6) | MICE |
| Weight (kg) | 2(0.8) | MICE |
| Comorbidities, n (%) |  |  |
| COPD | 0 | Complete |
| Coronary artery disease | 0 | Complete |
| Hypertension | 0 | Complete |
| Diabetes mellitus | 0 | Complete |
| Sepsis | 0 | Complete |
| Vital signs |  |  |
| Respiratory rate (/min) | 0 | Complete |
| Heart rate (/min) | 0 | Complete |
| Systolic blood pressure (mmHg) | 14(5.6) | MICE |
| Diastolic blood pressure (mmHg) | 8(3.5) | MICE |
| SOFA | 0 | Complete |
| Laboratory data |  |  |
| Red blood cell (*10^12^) | 0 | Complete |
| White blood cell (*10^9^) | 0 | Complete |
| Platelet (*10^9^) | 1(0.4) | MICE |
| Hemoglobin (g/L) | 0 | Complete |
| Hematocrit (%) | 1(0.4) | MICE |
| Total bilirubin (mg/dL) | 0 | Complete |
| Alanine aminotransferase (U/L) | 4(1.6) | MICE |
| Aspartate aminotransferase (U/L) | 4(1.6) | MICE |
| Lactate dehydrogenase (U/L) | 22(8.8) | MICE |
| Sodium (mmol/L) | 0 | Complete |
| Potassium (mmol/L) | 0 | Complete |
| Calcium (mmol/L) | 0 | Complete |
| Glucose (mmol/L) | 0 | Complete |
| Creatinine (mg/dL) | 0 | Complete |
| Blood urea nitrogen (mmol/L) | 0 | Complete |
| Albumin (g/dL) | 20(8.0) | MICE |
| Prothrombin time (seconds) | 5(2.0) | MICE |
| APTT (seconds) | 4(1.6) | MICE |
| INR | 5(2.0) | MICE |
| Arterial pH | 0 | Complete |
| PaO_2_ (mmHg) | 0 | Complete |
| PaCO_2_ (mmHg) | 0 | Complete |
| Lactic acid (mmol/L) | 0 | Complete |
| Invasive ventilator, n (%) | 0 | Complete |
| Vasopressor, n (%) | 0 | Complete |
| Acute kidney injury, n (%) | 0 | Complete |
| CRRT, n (%) | 0 | Complete |

**Abbreviations:** MICE, Multiple Imputation by Chained Equations; COPD, Chronic obstructive pulmonary disease; SOFA, Sequential Organ Failure Assessment; APTT, Activated partial thromboplastin time; INR, International normalized ratio; PaO_2_, arterial oxygen partial pressure; PaCO_2_, arterial carbon dioxide partial pressure; CRRT, Continuous renal replacement therapy.

**Supplementary Table S2.** Bootstrap validation of the RF model.

| Metric | Value |
| --- | --- |
| Apparent AUC | 0.950 |
| Optimism | 0.027 |
| Optimism-corrected AUC | 0.923 |

**Abbreviations:** RF, random forest; AUC, Area Under the Receiver Operating Characteristic Curve.

**Supplementary Figure S1.** SHAP dependence plots showing the associations between age (A), lactate (B), RBC (C), and PO_2_ (D) and their corresponding SHAP values in the random forest model. Positive SHAP values indicate a higher predicted risk of in-hospital mortality. RBC, Red blood cell count; PO_2_, partial pressure of oxygen.
